# Supplementary material for: Identification of photoperiod-induced specific miRNAs in the adrenal glands of Sunite sheep (Ovis aries)
Source: Front Vet Sci. 2022 Jul 22;9:888207. doi: 10.3389/fvets.2022.888207 (PMC9354845; doi:10.3389/fvets.2022.888207)
Supplement: Supplementary file 1 [file Data_Sheet_1.ZIP › Supplementary file/miRDeep2 file.zip!/miRDeep2 file/Know_Structure/LP42A3/oar-mir-376c.pdf]

miRBase precursor : oar-mir-376c
Total read count : 735
oar-miR-376c-5p read count : 329
oar-miR-376c-3p read count : 405
remaining reads : 1

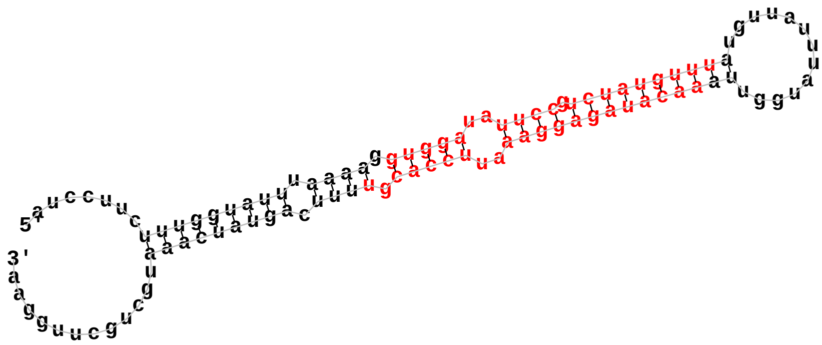

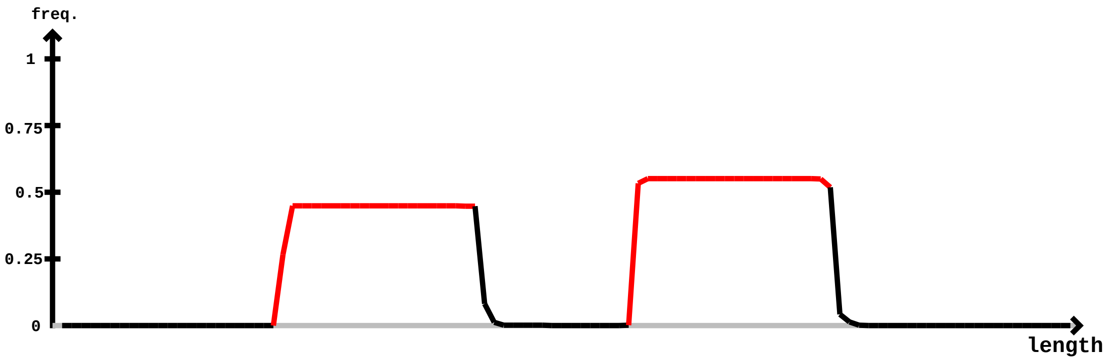


```
                                                                                   oar-miR-376c-3p
                        oar-miR-376c-5p
5'- auccuucuuugguauuuaaaagguggauauuccgucuauguuuauguuauuuaugguuaaacauagaggaaauuccacguuuucaguaucaaaugcugcuuggaa   -3'    exp
    .......(((((((((.((((.(((((..((((.((((((((((.............))))))))))))))..))))).)))).))))))))).............   reads    mm    sample
    ......................guggauauuccgucuaugu................................................................   1        0     seq
    ......................guggauauuccgucuauguuu..............................................................   165      0     seq
    ......................guggauauuccgucuauguuua.............................................................   27       0     seq
    ......................guggauauuccgucuauguuuau............................................................   3        0     seq
    .......................uggauauuccgucuauguuu..............................................................   104      0     seq
    .......................uggauauuccgucuauguuua.............................................................   24       0     seq
    .......................uggauauuccgucuauguuuau............................................................   5        0     seq
    .......................uggauauuccgucuauguuuauguuau.......................................................   1        0     seq
    ..........................................................aaacauagaggaaauuccacgu.........................   1        0     seq
    ...........................................................aacauagaggaaauuccac...........................   1        0     seq
    ...........................................................aacauagaggaaauuccacg..........................   23       0     seq
    ...........................................................aacauagaggaaauuccacgu.........................   349      0     seq
    ...........................................................aacauagaggaaauuccacguu........................   16       0     seq
    ...........................................................aacauagaggaaauuccacguuu.......................   2        0     seq
    ............................................................acauagaggaaauuccacguu........................   5        0     seq
    ............................................................acauagaggaaauuccacguuu.......................   7        0     seq
    ............................................................acauagaggaaauuccacguuuu......................   1        0     seq
```
